# Identifying High‐Risk Groups for Alzheimer's Disease Using Deep Embedded Clustering in Wisconsin Registry for Alzheimer's Prevention Participants

**DOI:** 10.1002/alz70856_105280

**Published:** 2026-01-07

**Authors:** Coco Victoria Gomez Tirambulo, Simona Merlini, Mithun Paul, Carlos Lizarraga, Roberta Diaz Brinton, Francesca Vitali

**Affiliations:** ^1^ University of Arizona, Tucson, AZ, USA

## Abstract

**Background:**

Individuals in the early stages of Alzheimer's disease (AD) constitute a heterogeneous group, with diverse risk factor profiles such as chromosomal sex, apolipoprotein E (APOE) genotype, and comorbidities, evolving over distinct time courses. Within a prodromal phase that can extend for one to three decades, opportunities and challenges exist in identifying crucial tipping points in progression and opportunities for prevention.

**Method:**

Our study aimed to identify subgroups within the 389 individuals at high‐risk for AD (65.6±6.4 years old, 67.1% female, 38.8% APOE ε4 carriers) from the Wisconsin Registry for Alzheimer's Prevention data, 2001‐2022. We analyzed prospectively collected data covering patient characteristics (age, sex, race, and APOE ε4 carrier status), medical history (history of diabetes, hypertension, and hyperlipidemia), plasma biomarkers (amyloid‐β (Aβ) 40, Aβ42, Aβ40/42 ratio, phosphorylated tau (*p*‐tau) 181, and *p*‐tau 217), and blood laboratory parameters (insulin, glucose, triglycerides, low‐density lipoprotein cholesterol, and high‐density lipoprotein cholesterol). Employing classical clustering methodologies (CCMs, k‐means (KMs), KMs with principal component analysis, hierarchical clustering (HC), and HC with dynamic time warping) to inform the unsupervised deep embedded clustering (DEC) algorithm, we evaluated cluster membership and assessed clinical validity. Variable contributions to the predicted cluster membership were assessed using SHapley Additive exPlanations values.

**Result:**

Our DEC findings demonstrated promising results by identifying more distinct risk profile patterns for each cluster (*n* = 6) compared to CCMs (*n* = 2); achieving a more evenly distributed partitioning of participants into clusters with increased stability, measured by Jaccard and entropy scores; and validating the clinical recognizability based on laboratory values, plasma biomarkers, physician cognitive diagnoses, and Preclinical Alzheimer Cognitive Composite scores. Cluster characterization revealed participants in cluster 6 (*n* = 44) were most at‐risk for AD, consisting of female APOE ε4 carriers with elevated *p*‐tau levels. Conversely, cluster 4 (*n* = 57) was the least at‐risk, youngest cluster, comprising females with fewer comorbid conditions and the lowest AD biomarker levels. Cluster 3 (*n* = 81) represented the control population.

**Conclusion:**

Going forward, these outcomes will enable a robust pipeline for integrating electronic medical record data, empowering diverse patient characterization, and better identify those at risk to implement personalized preventative treatment within heterogeneous populations at risk for AD.

